# A SCARECROW-RETINOBLASTOMA Protein Network Controls Protective Quiescence in the Arabidopsis Root Stem Cell Organizer

**DOI:** 10.1371/journal.pbio.1001724

**Published:** 2013-11-26

**Authors:** Alfredo Cruz-Ramírez, Sara Díaz-Triviño, Guy Wachsman, Yujuan Du, Mario Arteága-Vázquez, Hongtao Zhang, Rene Benjamins, Ikram Blilou, Anne B. Neef, Vicki Chandler, Ben Scheres

**Affiliations:** 1 Department of Molecular Genetics, Utrecht University, Utrecht, The Netherlands; 2 Laboratorio Nacional de Genmica para la Biodiversidad, Cinvestav Sede Irapuato, Irapuato, Mexico; 3 Instituto de Biotecnología y Ecología Aplicada (INBIOTECA), Universidad Veracruzana, Xalapa, Veracruz, Mexico; 4 Institute of Organic Chemistry, University of Zurich, Zurich, Switzerland; 5 BIO5 Institute and Department of Plant Sciences, University of Arizona, Tucson, Arizona, United States of America; University of California, San Diego, United States of America

## Abstract

Quiescent long-term somatic stem cells reside in plant and animal stem cell niches. Within the Arabidopsis root stem cell population, the Quiescent Centre (QC), which contains slowly dividing cells, maintains surrounding short-term stem cells and may act as a long-term reservoir for stem cells. The RETINOBLASTOMA-RELATED (RBR) protein cell-autonomously reinforces mitotic quiescence in the QC. RBR interacts with the stem cell transcription factor SCARECROW (SCR) through an LxCxE motif. Disruption of this interaction by point mutation in SCR or RBR promotes asymmetric divisions in the QC that renew short-term stem cells. Analysis of the *in vivo* role of quiescence in the root stem cell niche reveals that slow cycling within the QC is not needed for structural integrity of the niche but allows the growing root to cope with DNA damage.

## Abstract 2

Ben Scheres and colleagues report that in the growing tip of plant roots, a gene regulatory network that includes the plant homologue of Retinoblastoma regulates the divisions of long-term stem cells to replenish tissue and to protect the root stem cell niche.

## Author Summary

In the plant *Arabidposis thaliana*, root meristems (in the growing tip of the root) contain slowly dividing cells that act as an organizing center for the root stem cells that surround them. This centre is called the quiescent centre (QC). In this study, we show that the slow rate of division in the QC is regulated by the interaction between two proteins: Retinoblastoma homolog (RBR) and SCARECROW (SCR), a transcription factor that controls stem cell maintenance. RBR and SCR regulate quiescence in the QC by repressing an asymmetric cell division that generates short-term stem cells. Here we genetically manipulate the cells in the QC to alter their quiescence by regulating the RBR/SCR interaction to demonstrate that quiescence is not needed for the organizing capacity of the QC but instead provides cells with a higher resistance to genotoxic stress, allowing stem cells in the QC to survive even if more rapidly cycling stem cells are damaged. A role for mitotic quiescence has been reported in animal stem cells, in which Rb has been implicated. These findings indicate that it might serve a similar role in plant stem cells.

## Introduction

The development of multicellular organisms depends on the ability of stem cells to self-renew and to generate new cellular progeny. Transcription factors play key roles in the maintenance of the stem cell state. In embryonic stem cells, for example, stem cell transcription factors repress lineage-specific differentiation programs while maintaining cell proliferation [1],[2]. In mammals, stem cell quiescence occurs in multiple tissue contexts, where some cells divide infrequently but can recover multiple lineages after injury to the niche [3]–[5]. It has been proposed that quiescent cells reside alongside active stem cells to ensure longevity and output of stem cell compartments [6]. Although quiescence can be released, for example, in murine hematopoietic stem cells by the silencing of p21 [7] and Retinoblastoma (Rb) homologs [8], or by genetic ablation of active stem cells in the gut [5], it has been difficult to directly test how quiescent and active stem cells share labor within compartments.

Stem cell niches in plants and animals display structural similarities [9]. Active stem cells in the Arabidopsis root stem cell niche, also called initials, surround infrequently dividing quiescent centre (QC) cells (Figure S1A). The Arabidopsis QC is required to maintain division and prevent differentiation in the surrounding stem cells through non-cell-autonomous signaling [10],[11]. The central QC cells in Arabidopsis were initially identified as cells that infrequently enter S-phase measured by ^3^H-thymidine incorporation [12]. Later studies identified the QC as an organizing centre that signals to the surrounding stem cells to prevent differentiation [13],[14]. It has been proposed that the Arabidopsis QC also acts as a reservoir to replace short-lived stem cells in the root [13]–[14]. However, more than half a century since the initial description of the QC in maize roots [15], a thorough description of the division of labor between initials and QC cells, and a further investigation of the significance of QC quiescence, has been lacking.

In the Arabidopsis stem cell niche, two genetic pathways are involved in stem cell specification. One is dependent on the plant hormone auxin and the PLETHORA (PLT) transcription factor family [16]. The other involves protein movement and activation of the heterodimeric transcriptional regulator SCARECROW-SHORTROOT (SCR-SHR) [16]–[19]. In addition, reduction of the RETINOBLASTOMA-RELATED protein (RBR), the single Rb homolog, expands stem cell lineages in roots [20] and leaves [21]. SCR-SHR and RBR interact, and the resulting genetic network is biased by auxin and cell cycle progression in order to specify asymmetric cell division in the ground tissue stem cell [22].

Here, we show *in vivo* that QC cells, in addition to their role as niche organizer, replenish a distal stem cell pool. Intriguingly, quiescence and asymmetric cell division in the QC are balanced by RBR-SCR interactions, which also control asymmetric cell division in ground tissue stem cells. We provide evidence that the physiological function of quiescence is to control a trade-off between genotoxic stress protection and replacement of short-term stem cells.

## Results

### The QC Slowly Replenishes Columella Stem Cells

Previous clonal analyses revealed that in a WT root the QC divides, although at a low rate, and that the QC could be a source for all stem cells in the Arabidopsis root [23]–[25]. However, due to the low QC division frequency, their exact frequency and division pattern has not been determined.

**Figure 1:**
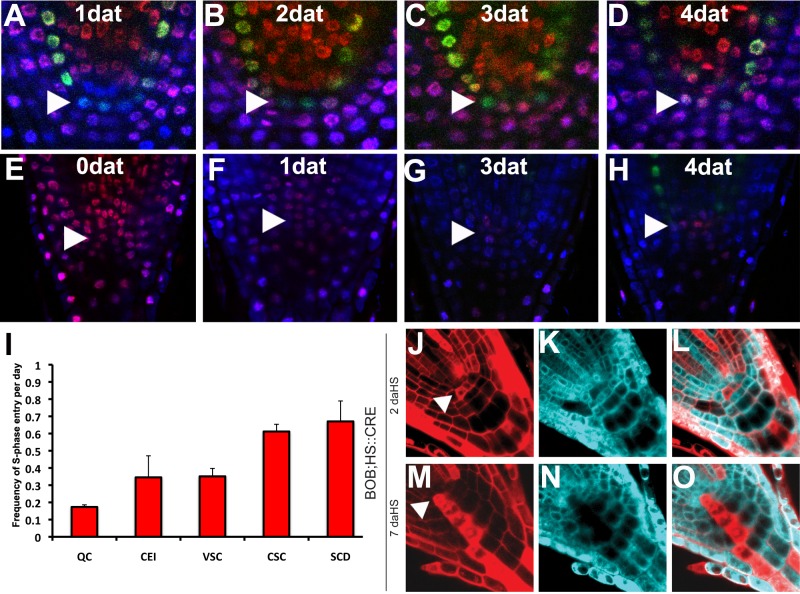
WT QC cells divide infrequently and incorporate into the columella. (A–D) 5 dpg *pSCR::SCR:GFP* plants showing S-phase label incorporation by F-*ara*-EdU for 1–4 dat. DAPI is shown in blue, F-*ara*-EdU staining is shown in red, and *pSCR::SCR:GFP* in green. (E–H) Pulse and chase experiment, plants were grown for 5 dpg in F-*ara*-EdU nucleoside analog, then transferred for 0–4 d into nonsupplemented medium. Arrowheads point to QC. (I) Quantification of entry in S-phase frequency for each cell type. QC, quiescent centre; CEI, cortex-endodermis initial; VSC, vascular stem cell; CSC, columella stem cell; SCD, stem cell daughter. (J–O) Confocal images of root meristems with a single WT BOB clone at 2 (J to L) and 7 (M to O) daHS. The 2 daHS single cell QC clone (arrow head in J) divided and its daughter cell incorporated to the columella region at 7 daHS (M). See also Figure S2.

We monitored entrance into S-phase using the nontoxic nucleoside analog F-*ara*-EdU [26], which allowed normal root growth for as long as 7 days after transfer (dat) (unpublished data). Time course analysis of F-*ara*-EdU uptake showed different times for entry into S-phase for each cell type (Figure 1A–D). In addition, plants were germinated in F-*ara*-EdU for 5 d and then transferred into nonlabeled growth medium. In such pulse-chase experiments, loss of the label could be detected from the transit amplifying area at 1 dat, but stem cells and QC could maintain label after 3–7 d (Figure 1E–H). Quantification of the data revealed that QC cells divide with half the frequency of the surrounding stem cells and one-fourth the frequency of transit amplifying cells (Figure 1I).

To assess the fate of the QC progeny, we used the BOB clonal analysis system, which allowed us to follow genetically marked QC cells and their progeny over time [27]. Briefly, clones generated by this system express two different fluorescent proteins depending on the recombination events, which allows for finer dissection of the clones. We heat-shock-induced 32 QC clones and followed them from 2 to 16 days after heat shock (daHS). Eighty-four percent (27 clones) of the QC clones (Figure 1J–L) divided during the experiment, and the rootward daughter always contributed to the columella (Figure 1M–O). We did not observe a contribution of any QC clones to the vascular and ground tissues. Our data indicate that, under physiological conditions, the QC undergoes infrequent divisions to populate the columella region.

### amiGO-Mediated Cell-Type-Specific RBR Knockdown and Complementation

**Figure 2:**
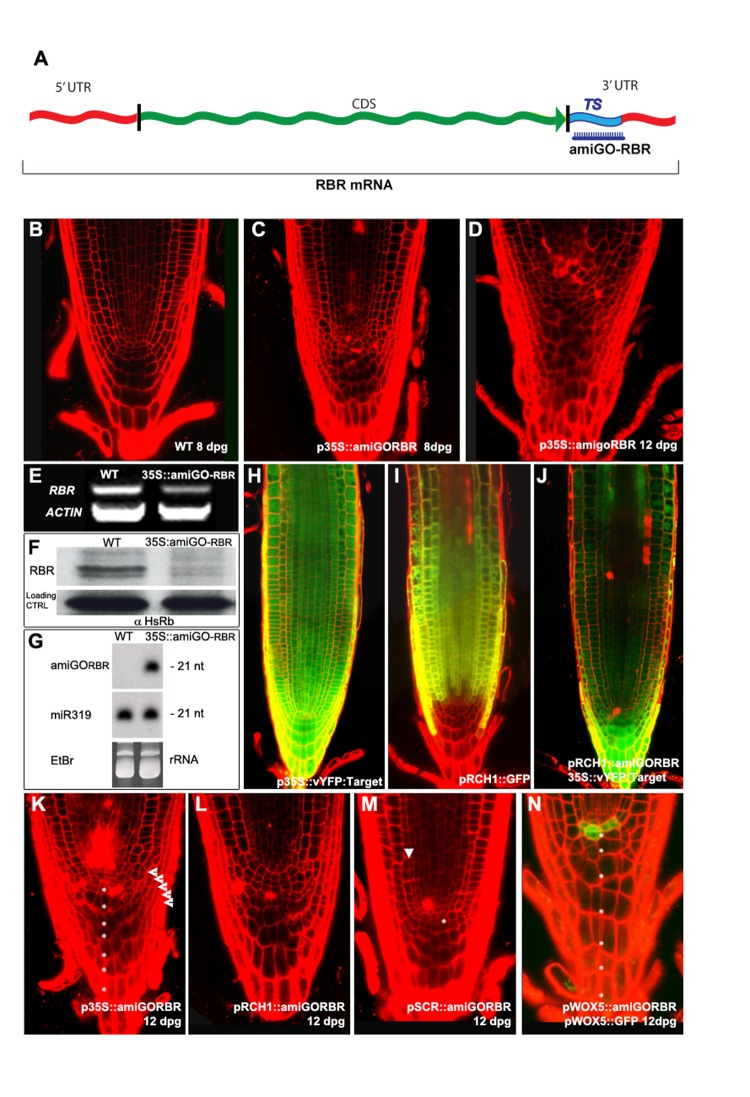
The AmiGO concept for RBR silencing. The AmiGO strategy. TS, Target sequence (A). Root apical meristem (RAM) of WT (B), *35S:amiGORBR* at 8 (C) and 12 (D) dpg seedlings. Validation of the amiGORBR RNA interference by RT-PCR detection of endogenous RBR transcripts (E) and Western blot analyses for RBR protein levels (F). Mature amiGO-RBR synthesis detected by small-RNA Northern blot (G). The *in planta* action of amiGO-RBR was revealed by introducing the sensor construct *35S::vYFP:amiGORBR-TS* in WT (H) and in the *pRCH1::amiGORBR* (J) backgrounds. Expression pattern of the *pRCH1::GFP* marker in the WT background (I) [54]. AmiGORBR expression driven by different promoters generates distinct phenotypes in primary root meristems of 12 dpg seedlings. (K) *p35S::amiGORBR* causes overproliferation of QC, LRC (arrow heads), and CSC (asterisks) as well as cell death in vascular and columella cells. (L) *pRCH1::amiGORBR* shows overproliferation of the QC and LRC and cell death and (M) *pSCR::amiGORBR* shows extra periclinal divisions of the ground tissue (arrow heads) and extra division of the QC (asterisks), while (N) *pWOX5::amiGORBR;pWOX5::GFP* shows QC divisions that cause an increase in columella layers (asterisks). See also Figures S3, S4, S5.

To relax quiescence in the stem cell niche, we developed a cell-type-specific RBR silencing tool based on artificial microRNA (amiRNA), termed artificial microRNA for Gene-silencing Overcome (amiGO) (Figure 2A), which allows for cell autonomous silencing and easy complementation (Text S1). When expressed ubiquitously, *amiGORBR* caused supernumerary divisions in stem cells, producing extra columella and Lateral Root Cap (LRC) layers that increased over time (Figure 2B–D), and phenocopying previously described roots with reduced RBR function [20],[27]. amiRNA accumulation was correlated with a reduction in RBR mRNA levels and decrease in protein levels (Figure 2E–G), and the degradation of the target was spatially constrained when amiRNA was driven from tissue-specific promoters (Figure 2H–J).

The phenotypes in the stem cell region were similar to those observed upon clonal deletion of *RBR* [27], indicating that the level of silencing was sufficient to deplete RBR function in these cell types. Promoters from the ground-tissue- and QC-expressed gene *SCR* and the QC-specific *WOX5* gene (Figure S1B–C) allowed us to investigate the role of RBR in specific cell types. In *pSCR::amiGORBR* roots, extra periclinal cell divisions occurred in the endodermis, consistent with the RBR role in this asymmetric cell division (Figure 2M, arrowhead) [22], and QC cells divided, while no extra LRC layers were produced (Figure 2K–M, *n* = 15). *pWOX5::amiGORBR* roots displayed extra QC divisions, shown by the presence of *pWOX5::GFP* marker in newly divided cells. In addition, the number of cell layers in the columella increased (Figure 2N, asterisks; *n* = 15). The LRC and ground tissue were not affected, consistent with a cell-autonomous role for *RBR* in QC maintenance.

WT plants had a maximum of two undifferentiated columella layers, but *p35S::amiGORBR* roots displayed up to four layers as revealed by starch granule staining. Quantification of the number of columella and LRC layers revealed that the increase in columella layers in *p35S::amiGORBR* roots was caused by extra divisions in both QC and columella stem cells, with each of the divisions creating one extra layer (Figure S3). These observations indicated that the rootward daughters of QC divisions contributed to the columella root cap.

To analyze the effect of RBR loss by a different strategy, we next induced and followed QC clones that lost at least one genomic copy of *RBR*. Homozygous BOB-RBR seedlings (*rbr-3/rbr-3;BOB-RBR^+/+^,WOX5::CRE:GR*) [27] were germinated on dexamethasone to induce *RBR* deletion clones in the QC. QC clones were selected prior to QC division (Figure S5A–C), and followed through division and differentiation. The rootward-most cells (Figure S5D–I) acquired starch granules characteristic of differentiated columella cells, demonstrating that QC cells with reduced RBR activity, as in the WT, contribute to the columella.

### RBR Represses Asymmetric Cell Division in the QC

**Figure 3:**
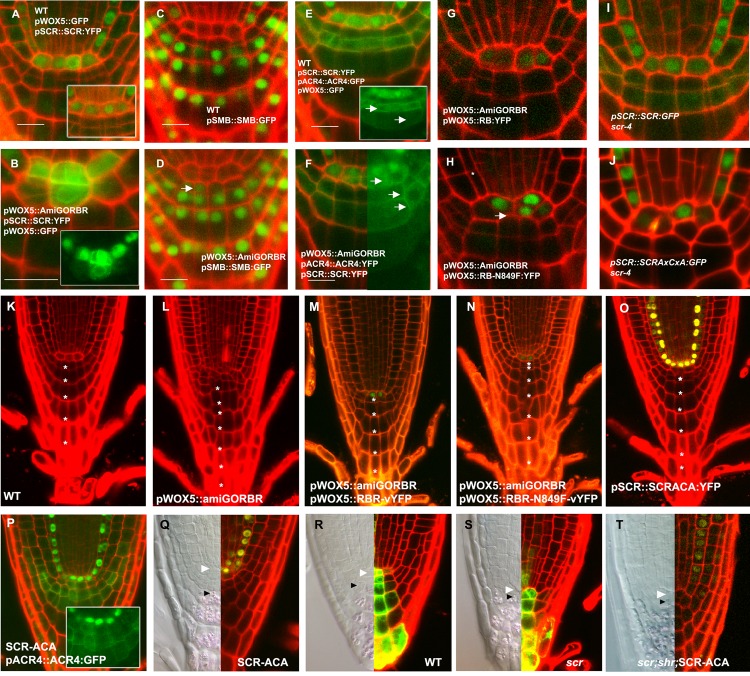
*RBR* silencing induces asymmetric cell division of the QC. Expression patterns of cell-fate markers in stem cell niche of WT (A, C, and E) and *pWOX5:amiGORBR* (B, D, and F) 5 dpg seedlings. Marker genotypes: cytoplasmic *pWOX5:GFP* and nuclear *pSCR:SCR:YFP* (A and B), *pSMB::SMB:GFP* (C and D), cytoplasmic *pWOX5:GFP*; nuclear *pSCR:SCR:YFP* and membrane *pACR4:ACR4:GFP* (E and F). Arrows in (D) indicate shift in SMB expression, and arrows in (E) and (F) point to plasma membrane-localized ACR4-GFP expression. Full rescue of the QC division in *pWOX5:amiGORBR* complemented with *pWOX5:RBR:YFP* and *scr-4* complemented with *pSCR::SCR:YFP* (G, I) and no rescue by LxCxE mutant *pWOX5:RBR^N849F^-YFP or pSCR::SCR^AxCxA^:YFP* (H, J). Root phenotype of 12 dpg seedlings of Col-0 WT (K), *pWOX5::amiGORBR* (L), *pWOX5::amiGORBR;pWOX5::RBR:vYFP* (M), *pWOX5::amiGORBR;pRBR::RBR^N849A^:vYFP* (N), and *pSCR::SCR^AxCxA^:YFP, scr-4* (O). Asterisks depict the columellla layers rootwards from the layer in contact with the QC and excluding the detaching distal layers. Production of extra columella stem cell in *pSCR::SCR^AxCxA^:YFP, scr-4* as shown with ACR4-GFP marker (P). Number of columella stem cell layers by lugol staining in *pSCR::SCR^AxCxA^:YFP, scr-4* (Q), Col-0 WT (R), *scr-4* (S), and *pSCR::SCR^AxCxA^:YFP shr-2 scr-4 plants* (T). See also Figure S6.

To address whether QC cell divisions were symmetric or asymmetric, we first confirmed the expression of *pWOX5::GFP* (ER fluorescence) and *pSCR::SCR:YFP* (nuclear fluorescence) in the undivided QC of WT (Figure 3A). After a QC cell divided in the *pWOX5::amiGORBR* background, both daughters expressed *pWOX5::GFP* (Figure 3B). However, the rootward daughter lost *pWOX5::GFP* signal over time (Figure S6A–C). *pSCR::SCR:YFP* was more rapidly lost in the rootward daughter but retained in the shootward daughter (Figure 3B), which, based on these markers, retained QC fate. To determine the fate of the rootward cell, we introgressed two columella markers, *pSMB::SMB:GFP* and *pACR4::ACR4:GFP*, marking one differentiated columella layer [28], and the plasma membrane of columella stem cells and daughters [29], respectively (Figure 3C–F). In *pWOX5::amiGORBR* roots, SMB-GFP was expressed in the cell bellow the divided QC cell (Figure 3C–D), and ACR4-GFP was expressed in the rootward daughter and two additional layers of columella (Figure 3E–F), indicating columella identity of the rootward cell. Time lapse analysis of dividing QC cells from 4 to 8 dpg using a brighter nuclear-localized *pACR4::H2B:YFP* reporter in the *pWOX5::amiGORBR* background confirmed the progressive acquisition of pACR4 promoter activity in the rootward daughter of the divided QC cell (Figure S6D–F). Together, our results reveal that reduction of RBR activity triggers more frequent asymmetric cell division (ACD) in the QC. This ACD generates one shootward daughter cell with QC fate expressing SCR and WOX5 and a rootward columella stem cell expressing ACR4.

### Asymmetric Division Repression by LxCxE-Motif–Dependent SCR Interaction

Binding of mammalian Rb to LxCxE-motif–containing proteins has been implicated in the maintenance of quiescence in animal cells [30],[31]. We therefore mutated RBR in residue 849 (RBR^N849F^), which has been implicated in the interactions between Rb and LxCxE motif proteins in animals [32]. Accordingly, in a yeast two-hybrid assay, the RBR^N849F^ mutant lost the capacity to interact with the strong LxCxE-dependent interactor Histone Acetyl-transferase 2 (HAT2), while it retained the capacity to bind E2Fa, which does not contain LxCxE motif (Figure S8). We fused this *RBR^N849F^* mutant and the WT *RBR* cDNAs to the *vYFP* CDS, under the control of the *WOX5* promoter (*pWOX5::RBR^N849F^:vYFP, pWOX5::RBR:vYFP*). Because the amiGO system does not target cDNA variants lacking the 3′-UTR sequence, these constructs could be tested for complementation in the *pWOX5::amiGORBR* background.

*pWOX5::amiGORBR*;*pWOX5::RBR-vYFP* roots fully complemented the QC division phenotype, assessed both by absence of QC division (Figure 3G) and by number of columella layers (Figure 3K–N and Table 1). *pWOX5::RBR^N849F^:vYFP*, however, failed to complement the QC division phenotype of *pWOX5::amiGORBR* (Figure 3H,N and Table 1), suggesting that the role of RBR in controlling QC division is dependent on its capacity to bind LxCxE-domain–containing proteins and not through E2F repression.

**Table 1:** Quantification of columella cell layers in *pWOX5::amiGO-RBR* lines complemented with RBR^WT^ and RBR^N849F^ variants (minimum *n* = 9).

Background	Columella Layers (12 dpg)
*pWOX5::amiGORBR*	7.2±0.3
*pWOX5::amiGORBR/pWOX5::RBR-vYFP*	5.2±0.5
*pWOX5::amiGORBR/pWOX5::RBR^N849F^-vYFP*	6.6±0.3
Wild-type Col0	4.7±0.4

**Table 2:** Quantification of QC division frequency upon 1 µM Hydroxyurea treatment in Col-0, *pWOX5:amiGORBR*, and *pSCR::SCR^AxCxA^:vYFP*, *scr-4* (*n* = 20).

Background	HU Treatment	Frequency of QC Division
Col-0	0 h	0.18±0.03
	24 h	0.43±0.12
	48 h	0.62±0.07
*pWOX5::amiGORBR*	0 h	0.47±0.16
	24 h	0.59±0.04
	48 h	0.62±0.05
*pSCR::SCR^AxCxA^:YFP scr-4*	0 h	0.65±0.07
	24 h	0.78±0.06
	48 h	0.76±0.22

**Table 3:** Quantification of root length upon zeocin treatment in Col0, *pWOX5:amiGORBR*, and *pSCR::SCR^AxCxA^:vYFP*, *scr-4*.

Background	Average Root Length	Standard Deviation	Student *t* test	*n*
WT-24 hpz	10.6	0.9		45
WT-48 hpz	13.9	1.2		45
WT-72 hpz	15.8	1.3		45
*pWOX5:amiGO*-24 hpz	11.0	0.8	0.165158982	20
*pWOX5:amiGO*-48 hpz	12.0	1.2	1.06026E-07	20
*pWOX5:amiGO*-72 hpz	12.3	1.2	6.64334E-15	20
*pSCR::SCR^AxCxA^:YFP*-24 hpz	10.7	1.1	0.854187792	27
*pSCR::SCR^AxCxA^:YFP*-48 hpz	12.9	1.3	0.00150098	27
*pSCR::SCR^AxCx^A:YFP*-72 hpz	13.4	1.2	3.27424E-11	27

It has recently been shown that RBR represses SCR activity via LxCxE binding to inhibit asymmetric cell division in the mature endodermis tissue [22]. As SCR is involved in establishing and maintaining QC identity, we analyzed the role of the RBR-SCR interaction in QC division, by studying *pSCR::SCR^AxCxA^:YFP; scr-4* mutants, where the RBR-SCR interaction is abolished [22]. Notably, *pSCR::SCR^AxCxA^:YFP; scr-4* mutants displayed extra QC divisions and one extra columella layer (Figure 3I–J,O), indicating that the regulation of QC cell division by RBR is likely due to its interaction with SCR.

**Figure 4:**
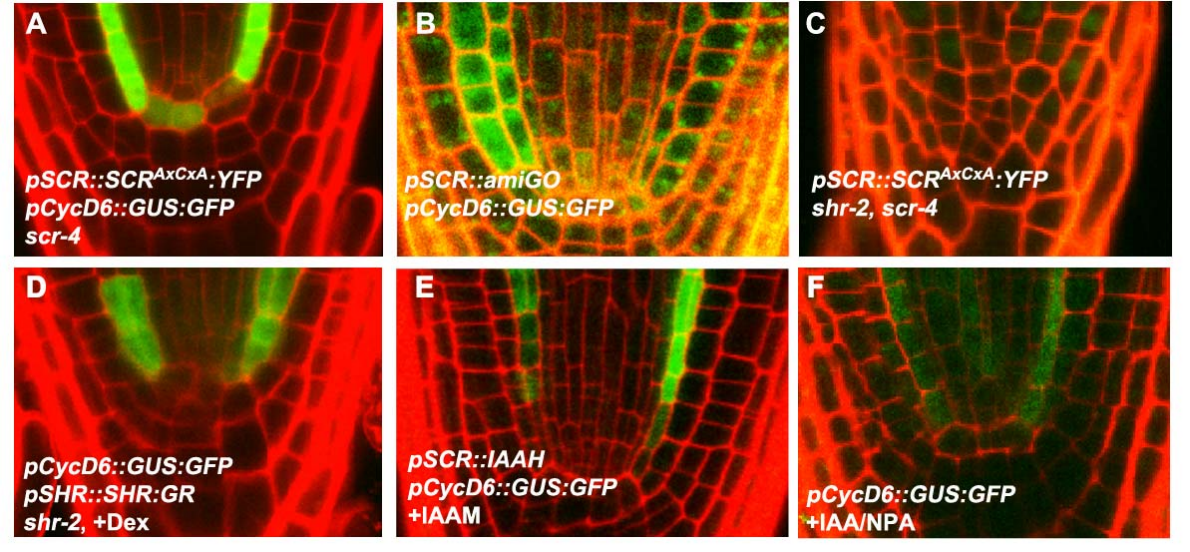
A modified RBR-SCR network in the QC. (A) *pCycD6::GUS:GFP* expression in QC of *pSCR::SCR^AxCxA^:YFP, scr-4* background. (B) Down-regulation of RBR in the pSCR domain in *pSCR::amiGORBR* fails to induce pCycD6 transcription in the QC. (C) *pSCR::SCR^AxCxA^:YFP* does not recover QC division in *scr-4*, *shr-2* background. (D) *pCycD6::GUS:GFP* expression upon SHR induction in *pSHR::SHR:GR*, *shr-2* background only in ground tissue and not in QC cells. (E) *pCycD6::GUS:GFP* expression in *pSCR::IAAH* line, treated with IAAM; note induction in endodermis but not in QC. (F) Auxin accumulation does not induce CycD6 transcription in the QC.

In the endodermis, SCR and SHR activate CycD6;1 expression, which in turn phosphorylates RBR and inhibits RBR-mediated inactivation of the SHR-SCR complex [22]. However, CycD6;1 was very weakly expressed in the QC of *pSCR::SCR^AxCxA^:YFP; scr-4* plants and *pSCR::amiGORBR* plants (Figure 4A,B). There was no QC division in the *pSCR::SCR^AxCxA^:YFP; scr-4, shr-2* background (Figure 4C), demonstrating that, as in ground tissue, this division depends on SHR. Together, these data indicate that QC division is restrained by the SHR-SCR-RBR network.

Induction of SHR activated CycD6;1 expression in the ground tissue but not in the QC (Figure 4D). Auxin, either endogenously produced in the QC and ground tissue layer (Figure 4E) or exogenously applied (Figure 4F), was also not able to induce CycD6;1 expression in the QC at levels comparable to the ground tissue, nor could we detect more QC divisions upon increased auxin activity. Thus, a QC factor normally inhibits CycD6;1 transcription by the SHR-SCR circuit, and the QC division is not dependent on high CycD6;1 activity.

As our data showed a correlation between QC division rates and the number of columella layers, we analyzed the columella in mutants for RBR-SCR network components. Higher SCR-SHR activity led to extra layers (Figure 3Q), whereas lack of activity led to fewer layers in *scr-4* and *shr-2* mutant backgrounds (Figure 3R–T). Together, our data indicate that division of the QC is regulated by components of the network that regulates ground tissue stem cell ACD with the exception of CycD6;1. In the QC context, this network variant is used to regulate replenishment of the columella stem cell pool.

### QC Division Interferes with Recovery Upon Injury to the Niche

**Figure 5:**
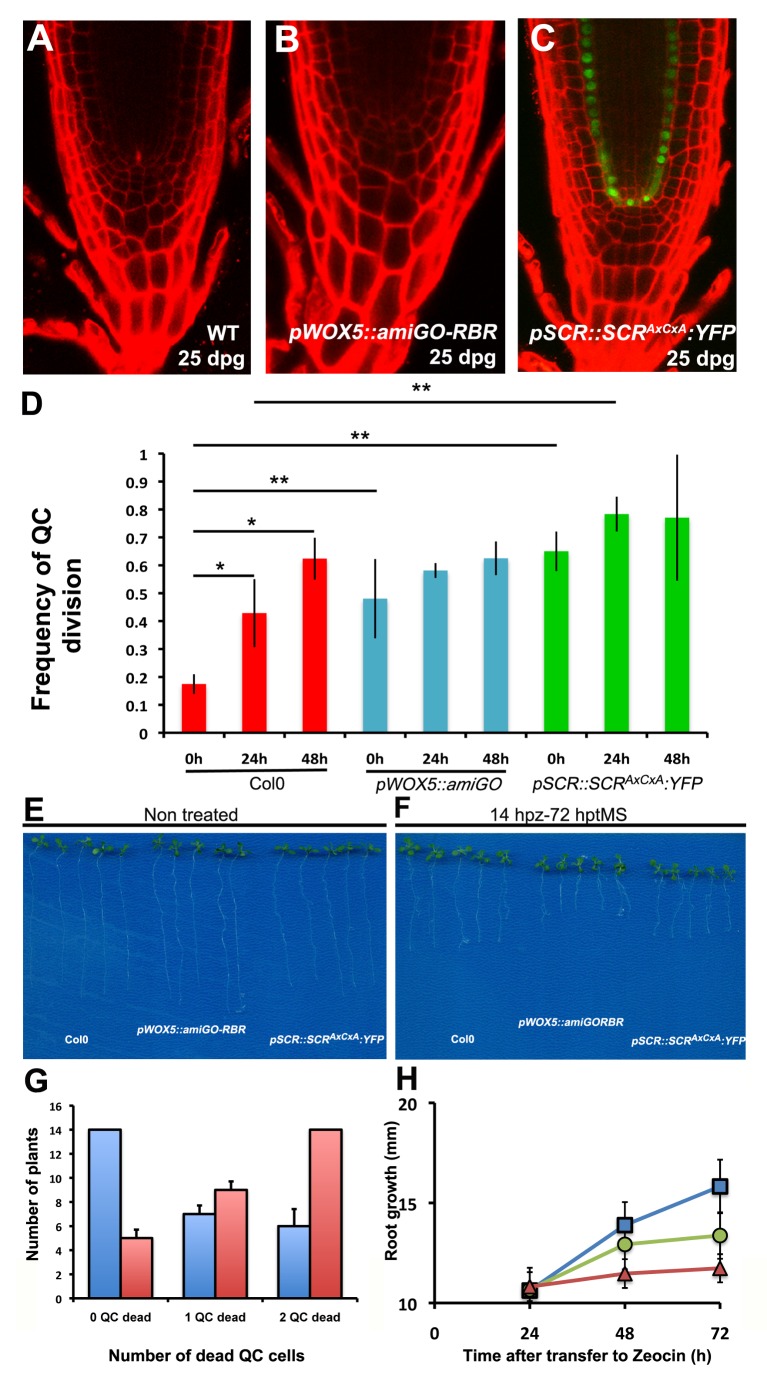
Dividing QC cells are more sensitive to DNA damage. (A–C) Root stem cell niches at 25 dpg in WT Col 0 (A), *pWOX5::amiGORBR* (B), and *pSCR::SCR^AxCxA^:YFP*, *scr-4* (C) plants. (D) Quantification of QC division frequency upon hydroxyurea treatment. Roots were analyzed for QC divisions after treatment with replication stress inducer HU. Statistical significance (*p*<0.05) using student's *t* test for treatments (*) and genotypes (**). (E–G) Effect of zeocin treatment in root growth. Primary root growth after genotoxic stress is impaired in *pWOX5::amiGORBR* and *pSCR::SCR^AxCxA^:YFP*, *scr-4* (E–F) proportionally to the number of dead QC (G); blue column shows number of WT plants analyzed for cell death and red shows *pWOX5::amiGORBR*. Quantification of root growth after zeocin treatments (H) in WT (blue squares), *pWOX5::amiGORBR* (red triangles), and *pSCR::SCR^AxCxA^:YFP*, *scr-4* (green circles). Student's *t* tests indicate statistical significance (*p*<0.05) of all differences between genotypes at 48 and 72 h. See also Figure S7 and Tables 2 and 3.

To address the significance of quiescence, we analyzed the effect of a faster dividing QC in root development. Root growth rates of *pWOX5::amiGORBR* plants that have continuously dividing QC cells are similar to those of WT at 10 and 25 dpg (unpublished data). Moreover, there were no evident effects on stem cell niche activity and organization in 25 dpg *pWOX5::amiGORBR* or *pSCR::SCR^AxCxA^:YFP; scr-4* roots (Figure 5A–C). These observations demonstrate that quiescence of the organizing cells is not strictly necessary for function or structural integrity of the niche over this time span, and that the shootward daughter after ACD retains full QC function in its continued ability to maintain the stem cell niche.

Replication stress is known to induce division in quiescent stem cells [33],[34]. We tested whether, in analogy, the induction of QC division might be induced by the ribonucleotide reductase inhibitor hydroxyurea (HU), which is known to delay S phase entry [35]–[37]. Indeed, treatment with 1 µM HU significantly increased the frequency of QC division in Col-0, but did not further increase QC division in *pWOX5::amiGORBR* and in *pSCR::SCR^AxCxA^:YFP; scr-4* plants. (Figure 5D). We concluded that replication stress enhances QC divisions through the SCR-RBR pathway.

HU causes cell death at higher concentrations [38],[39]. In addition, columella and vascular tissue stem cells in Arabidopsis roots, but not QC cells, undergo cell death after treatments with drugs that induce DNA damage [40]. In line with classical ideas on the function of mammalian stem cell quiescence, we hypothesized that the reduced mitotic activity of QC cells might enable them to escape cell death and thus ensure the permanence of the organizing centre in the long term and, as a consequence, the maintenance of the root stem cell niche. Therefore we asked whether actively dividing QC cells in *pWOX5::amiGORBR* roots respond as sensitively as short-term stem cells to DNA damage.

We first analyzed the response of WT, *pWOX5::amiGORBR* and *SCR::SCR^AxCxA^:YFP; scr-4* roots when grown in the presence of the DNA-damaging agent Zeocin. In line with previous observations [40], WT roots accumulate propidium iodide (PI) as a sign of cell death in the vasculature and columella stem cells (Figure S7A) after 14 hours postzeocin (hpz), and none of the plants analyzed showed QC death (*n* = 27). However, in 63% (21/33) of *pWOX5::amiGORBR* roots, at least one of the QC cells accumulated PI, in addition to vascular cells and columella stem cells (Figure 5G).

We next pulsed plants with Zeocin to analyze the effects of cell death on the proliferative capacity and growth potential of the roots. At 24 hpz, WT roots with intact QC decreased growth (Figure 5E–F). Importantly, *pWOX5::amiGORBR* roots with similar stem cell loss but additional QC cell death revealed an exaggerated root growth reduction as shown by the proximity of the root differentiation zone to the meristem and the induction of root hair formation and elongation near the root tip (Figure S7G–H), indicating cell division arrest and progressive differentiation.

At 72 hpz, WT roots showed a disorganized, but still active, meristem, while *pWOX5::amiGORBR* roots underwent rapid differentiation of the transit-amplifying cells followed by differentiation of the root meristem (Figure S7E–F), which is reflected in the reduction of primary root growth (Figure 5H).

Together, these data indicate that division of the QC is induced to replenish columella cells and restrained to create a differential stress response within the long-term stem cells that allows the root to cope with DNA stress.

## Discussion

Here we demonstrate that reduction of RBR levels leads to asymmetric cell divisions in the central QC cells of the stem cell niche, thus regenerating short-term stem cells. In addition, our data indicate that RBR acts in a cell-autonomous manner to maintain near-quiescence within the QC.

The QC was initially identified as a group of cells with a relatively low mitotic activity at the position where different cell files that form the root converge. Subsequently the QC has been shown to form the structural and functional core of the root meristem in diverse plant species [41]. In Arabidopsis, laser ablation of the QC leads to differentiation of surrounding stem cells [13]. This maintenance function cell-autonomously requires SCR [18]. However, the Arabidopsis QC can perform infrequent divisions, which become more abundant at elevated temperature [23]. These divisions are promoted by the plant hormones ethylene and brassinolide, and do not interfere with stem cell maintenance [24],[42]. Our results fit with these observations, since extra ACDs caused by the silencing of RBR in the QC do not affect maintenance of the stem cell niche and QC gene expression patterns.

It was previously proposed that QC divisions facilitate replacement of the stem cell pool, and therefore that the QC can be a source of stem cells of all lineages in the Arabidopsis root meristem [23],[42]. In contrast, our results show that after RBR silencing in the QC under normal growth conditions, using either the amiGO or BOB systems, QC divisions only produce columella stem cells. These observations suggest that the shared embryonic lineage of QC and columella may bias QC cells to acquire, upon SCR-mediated asymmetric cell division, columella fate rather than ground tissue fate. Upon stem cell niche injury, however, positional and hormonal cues are dominant over lineage cues in respecifying QC and stem cells from actively dividing cells [14],[43]. It is surprising to note that the SHR-SCR network, hitherto strictly correlated with ground tissue (cortex and endodermis) cell fates, controls two asymmetric cell divisions resulting in different cell identities, demonstrating that this pathway can be deployed for ACDs giving rise to different cell fates. We can at this point not exclude that, alternatively, the SCR-RBR complex represses a QC cell division specified by other factors.

It is thought that the quiescence of stem cells in animals is pivotal to ensure tissue maintenance and to protect the stem cell pool from exhaustion under diverse stresses (reviewed in [44]). In mammalian cells, cell cycle exit (also referred to as quiescence) is a key step during differentiation. Rb mutants disrupted in LxCxE binding in mammalian cells exhibit defects in quiescence, both during differentiation of myocytes mediated through HDAC1 [32] and during stress-induced senescence mediated by chromatin remodeling proteins such as RBP1, Sin3, CtBP, HDAC1, HDAC2, and RbAp46, [45]. Our results suggest that interaction of Rb with LxCxE-containing proteins may represent an evolutionarily conserved mechanism for modulating quiescence.

Under natural growth conditions the plant root niche faces multiple biotic and abiotic stresses. Several of these stresses have been shown to affect stem cell niche maintenance [46],[47]. Additionally, environmental challenges such as hypoxia, temperature, or ozone stress can cause DNA damage, leading to cell death [48]. Similar pathways control cell cycle progression and the cell cycle window where cell differentiation and apoptosis can be initiated, and they seem to converge on RB function in mammalian cells during early G1 [49]. DNA stress caused by hydroxyurea or 5-fluorouracil induces division and differentiation in hematopoietic stem cells, leading to a premature loss of the stem cell niche. Exhaustion of the stem cell niche also occurs after loss of p21, which inhibits cyclin/cdk complexes that inactivate Rb, suggesting that Rb-related pathways also control quiescence and DNA damage. Loss of p21 leads to cell death upon treatment with hydroxyurea. In plants, where there is no p21, RBR inactivation leads to QC division, and the same thing happens upon hydroxyurea treatment. However, in plants the pathways do not seem to be additive as in mammalian cells, because hydroxyurea treatment of *pWOX5::amiGORBR* has no effect in QC division frequency. This suggests that the same pathway that activates QC division by hydroxyurea treatment is regulated by the RB-SCR network.

Intriguingly, Arabidopsis columella and vascular tissue stem cells are more sensitive than the QC to zeocin-induced DNA damage [40]. In a similar way, stem cell niches in epithelia contain two stem cell populations, of which the slow dividing population is able to replenish multiple lineages after injury [5]. Our data indicate that RBR-dependent quiescence of the QC plays a crucial physiological role in the maintenance of the niche, and maintains the QC cells as a stem cell reservoir. Quiescence does not need to be absolute in order to protect cells from DNA damage, but rather modest changes in cell cycle frequency are sufficient to bestow protection. Accordingly, shoot apical meristems of plants do not contain distinct QCs but rather a central zone undergoing slower cycling rates. While it is clear that activation of the QC division potential in the root can be triggered by stem cell damage, for example, by laser ablation [14] or stress signals [42], future work should reveal how exactly plants control the balance between protective quiescence and replacement of short-term stem cells.

## Materials and Methods

### Plant Growth Conditions

Seeds were fume sterilized in a sealed container with 100 ml bleach supplemented by 3 ml of 37% hydrochloric acid for 2–5 h, then suspended in 0.1% agarose, and plated on a growth medium consisting of half-strength Murashige Skoog salts, 1% sucrose, 0.8% plant agar, MES (pH 5.8), 50 mg/ml ampicillin, and 1–5 mM dexamethasone (optional for CRE∶GR induced clones), stratified for 2 d in 4°C dark room, and grown vertically in long day conditions (16 h light followed by 8 h of dark). For HS induction, plates with 2–3 days postgermination (dpg) seedlings were placed in a 37°C incubator for 1 h and analyzed 2 d later.

### Cloning of the amiRBR Precursor

The 21 mer amiGORBR (5′-UACAGAUGCUAUAACUGAGGA-3′) and amiGORBR* (5′-UACUCAGUUAU ACCAUCUGUA-3′) were cloned into Arabidopsis endogenous miR319a precursor via overlapping PCR. The final precursor for amiGORBR was amplifying using modified AttB1 and AttB2 primers and the PCR product was recombined by a BP Single Gateway reaction (Invitrogen) in a pGEM-T easy 221 vector for further use in Multisite Gateway Cloning (Invitrogen). The amiGORBR is antisense to the 21 nt sequence located at 3′UTR of RBR mRNA (5′-UCUUCAGUUAUAG CAUCUGUA-3′).

### AmiGO-RBR Northern Blot

We loaded 20 µg of the small RNA-enriched fraction per lane, and 5′-end-labeled oligonucleotide complementary to the mature amiGORBR was used as probe. The experiment was performed as described [50].

### RT-PCR Analysis

Total RNA of Col-0, *p35S::amiGORBR* (Col-0), *pRB::gRB:GFP* (Col-0), and *p35S::amiGORBR;pRB::gRB:GFP* seedlings at 5 dpg were obtained using Spectrum Plant total RNA Kit (Sigma). The cDNA was synthesized from 1 µg total RNA using odT18VN primer (Biolegio) and RevertAid M-MuLV reverse transcriptase (Biolegio). For the PCR reaction, a 2 µl cDNA sample was used to amplify in a total volume of 20 µl. The relative expression levels of *RBR* and *RB:GFP* mRNAs were determined by using primers (Biolegio): RBR FW (5′-GATCAAAGATGGATGCTC-3′) and RBR RV (5′-TACAGATGCTATAACTGAAGA-3′) for *RBR*; RBR FW (5′-GATCAAAGATGGATGCTC-3′) and GFP RV (5′-GAATTGGGACAACTCCAG-3′) for *RB:GFP*. *ACTIN1* expression was determined as an internal control using primers Actin FW (5′-GCCGATGAAGCTC AATCCAAA-3′) and Actin RV (5′-GGTCACGACCAGCAAGATCAA-3′).

### Western Blot Analysis of RBR Expression Levels

For analysis of protein expression in planta, plants were grown for 12 d under long day conditions, and 0.5 g of roots were grinded and extracted in Extraction Buffer (100 mM Tris-HCl PH 7.5, 150 mM NaCl, 0.5% Nonidet P-40, 1 mM phenylmethylsulfonyl fluoride [PMSF], 2× Protease inhibitor cocktail, 100 µM MG132). Equal amounts of protein extracts were loaded in a gel and transfered to a Hybond-ECL membrane (GE Healthcare) and inmunodetected with anti-RB antibody (provided by Dr. L. Bako) 1/7,500 and goat-anti-chicken 1/20,000 (ab97131 Abcam) and developed with Amersham Western Blotting Detection Reagent (GE Healthcare).

### Destination Clones and Plant Transformation

Constructs and plant lines used are listed in Table S1. All different constructs using the amiGORBR expression (*p35S::amiGORBR*, *pRCH1::amiGORBR*, *pSCR::amiGORBR*, and *pWOX5::amiGORBR*) were generated using Multisite Gateway technology (Invitrogen). *CaMV 35S*-driven amiGORBR construct was generated using a pGII229 binary vector, while other promoter-specific versions were recombined into a pGII226 binary vector.

To generate an amiGORBR sensor line, a version of *Venus YFP* (*vYFP*) containing the amiGORBR target sequence at its 3′ was first obtained by nested PCR and recombined into a pGEM-T easy 221 entry vector. The *vYFP_amiRBRtarget_* fragment was then recombined into a pB7m34GW binary vector under the *CaMV 35S* promoter. Transformation was performed on Columbia ecotype Col-0 and transgenic *pWOX5::amiGORBR* (Col-0) plants according to the floral dip method [51]. The description of all constructs and lines generated for this study is listed in Text S1 and Table S1.

### Phenotype Analysis and Microscopy

Whole-mount visualization of roots and starch granule staining were previously described [52]. Starch granules in the columella root cap were stained with 1% lugol solution for 30 s before the visualization. Confocal laser scanning microscopy (CLSM) images were performed on a Leica SP2 inverted laser-scanning microscope. Analysis of BOB clones was performed as described [27].

### *RBR* Clonal Deletion Experiments

Construction and use of the BOB deletion system is described in Wachsmann et al. 2011 [27]. Seedlings harboring red or cyan clones were preselected under Leica MZ16F fluorescence stereoscope and further analyzed by confocal microscopy. To excite and collect red, cyan, and yellow fluorescences in a Leica SP2 confocal microscope, we performed sequential scanning as follows: the CyPet_ER_ and the vYFP_NLS_ were excited together using 458 and 514 nm laser, respectively, and emission was collected at 465–506 nm for the CyPet_ER_ and 523–566 nm for the vYFP_NLS_. Propidium iodide, which marks cell walls (3 mg/ml, final concentration), and TagRFP_ER_ were visualized by exciting at 488 nm and 543 nm, respectively, and emission collected at 502–522 and 561–633 nm.

### Yeast Two-Hybrid Assay

Interactions between RBR and HAT were analyzed by yeast two-hybrid using the ProQuest Two Hybrid System (Invitrogen Life Technologies). RBRwt, RBRN849F, E2Fa, and HAT sequences were cloned in pDONR221 and recombined into pDEST32 BD (former two) and pDEST22 AD (latter two). Yeast two-hybrid analysis was performed by duplicate as previously described [22].

### Drug Treatment

MS plates containing 0.5% phytagel were supplemented with 20 µg/ml zeocin (Duchefa Z0186). Plants were grown after transference for a minimum of 14 and a maximum of 24 h. Plants were analyzed using PI-staining and CLSM. For primary root growth analyses after zeocin, data shown are the results of two biological duplicates, with a minimum of 20 seedlings per line in each duplicate.

F-*ara*-EdU treatment was performed in MS plates containing 0.5% phytagel and supplemented with 2 µM F-*ara*-EdU, which was synthesized as described [26]. Incorporation treatments were performed by transfering 4 dpg seedlings to F-*ara*-EdU–containing plates and growing the plants for further 1–4 dat. Pulse and chase experiments were performed by germinating the seeds in F-*ara*-EdU–containing plates for 5 dpg and then transferring them into MS plates for further 1–4 dat. Plants were then fixed in 1% formaldehyde, 0.1% Triton X-100 in PBS, and Click-iT EdU staining kit (C10338, Invitrogen) was used for signal development before image analysis by confocal microscopy as previously described [53].

Hydroxyurea (HU) treatment was performed in MS-agar plates supplemented with 1 µM HU (SIGMA, H-8627). We treated 4 dpg seedlings for 24 and 48 h, and the root apical meristem of treated plants was analyzed by confocal imaging. QC divisions were scored as QC cells with a newly formed cell wall. Frequency analysis was performed from 20 roots in duplicate experiments. Statistical differences between treatments, as well as between genotypes, were assessed using pairwise student's *t* tests.

## Supporting Information

Figure S1 **Root meristem in Arabidopsis thaliana.** Different cell types in the root apical meristem of Arabidopsis thaliana. Quiescent center, QC; Columella Stem Cell, CSC; Columella differentiated, Col; Lateral Root Cap, LRC; Epidermis, Epi; Cortex, Cor; Endodermis, En; Vasculature, Vasc. Cortex and Endodermis comprise the ground tissue (green); the columella tissue is represented in orange, and QC cells are yellow. (A) SCR expression domain, in QC, ground tissue stem cells and endodermis (B), and WOX5 expression domain in QC (C).
(TIF)

Figure S2 **QC incorporates F-ara-EdU at longer times than surrounding stem cells.** Left images show red (F-ara-EdU) and blue (DAPI staining) channels; right pictures show overlayed green (*pSCR::SCR:GFP*) channel. Arrowhead shows QC region that is stained by *pSCR::SCR:GFP*. Note that all green nuclei have no F-ara-EdU signal at 1–3 dat, but they show signal at 4 dat. Root meristem shown (A–B) 1 dat, (C–D) 2 dat, (E–F) 3 dat, and (G–H) 4 dat.
(TIF)

Figure S3 **RBR down-regulation induces divisions in the QC and CSCs, leading to extra columella layers.** (A) In roots of 6 dpg WT seedlings, a single, or a dividing, CSC (blue colored) is present between the QC (green) and the first columella layer with starch granules (arrowhead). (B) In roots of 6 dpg *p35s::amiGO-RBR* seedlings, three layers of nondifferentiated columella cells (blue) are present between the QC (green) and the first columella layer with starch granules (arrowhead). (C) In roots of 8 dpg *p35s::amiGO-RBR* seedlings, divisions of the QC are observed (green) together with extra proliferation of nondiferentiated collumela cells (blue) above first columella layer with starch granules (arrowhead).
(TIF)

Figure S4 **amiGO RBR lines can be complemented by constructs lacking the ami-complementary region.** 12 dpg *p35S::amiGORBR* (A), complemented with *pWOX5::RBR:vYFP* (B) or *pRBR::RBR:vYFP* (C), show partial (B) and total (C) complementation of the *p35S::amiGORBR* phenotype (A).
(TIF)

Figure S5 **RBR-depleted QC divides and daughters differentiate as mature Columella.** CLSM images of a single root tip recorded at 2 (A–C), 4 (D–F), and 8 (G–I) dpg *rbr/rbr;BOB-RBR+/+;pWOX5::CRE:GR* germinated on Dex-containing medium. A single QC cell (A and C, TagRFPER marked clone) missing one or two RBR copies divides (D and F) and ultimately gives rise to differentiated columella cell marked by starch granules (G to I, three cells enclosed by a dashed line).
(TIF)

Figure S6 **WOX5 and ACR4 marker accumulation before and after QC division.** *pWOX5::GFP* expression (A to C) and *pACR4::H2B:YFP* (D to F) was monitored and recorded from day 4 until day 8 postgermination in dividing QCs of *pWOX5::amiGO-RBR* roots. Asterisks indicate shootward daughters and arrowheads point to rootward daughters.
(TIF)

Figure S7 **Zeocin effects in the root stem cell niche.** 5 dpg seedlings from Col0 and pWOX5::amiGO backgrounds were transferred to medium with or without Zeocin (40 µM) for 14 h (hpz), analyzed, then transferred back to MS medium (hptMS), and monitored at 24 to 72 hptMS CLSM images of root meristems of Col-0 WT (A, B, and G), *pWOX5::amiGORBR* (C, D, and H), and *pSCR::SCR^AxCxA^:YFP*, *scr-4* (E and F).
(TIF)

Figure S8 **RBR^N849F^ fails to interact with LxCxE-containing proteins.** Yeast two-hybrid analysis showing interaction between RBR and HAT2, RBR^N849F^ and E2Fa, and disruption of interaction between RBR^N849F^ and HAT2.
(TIF)

Table S1 **List of plant constructs generated in this study.** Plant material used in the study and resistance and reference information.
(DOCX)

Text S1 **Supplementary information.** Construction and testing of the AMIGO gene silencing system and supplemental references.
(DOC)

## Abbreviations

ACD: asymmetric cell division
amiRNA: artificial microRNA
amiGO: amiRNA for gene silencing overcome
BOB: brother of brainbow
dat: days after transfer
dpg: days postgermination
HU: hydroxyurea
LRC: lateral root cap
PLT: plethora
QC: quiescent center
RBR: retinoblastoma-related protein
SCR: Scarecrow
SHR: Shortroot
WT: wild type
