# Supplementary figures and images for: A SCARECROW-RETINOBLASTOMA Protein Network Controls Protective Quiescence in the Arabidopsis Root Stem Cell Organizer
Source: PLoS Biol. 2013 Nov 26;11(11):e1001724. doi: 10.1371/journal.pbio.1001724 (PMC3841101; doi:10.1371/journal.pbio.1001724)

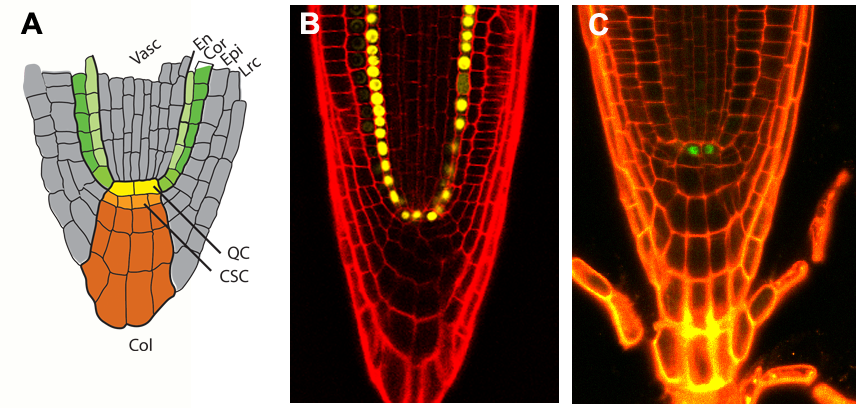

Supplement: Figure S1 — Root meristem in Arabidopsis thaliana. Different cell types in the root apical meristem of Arabidopsis thaliana. Quiescent center, QC; Columella Stem Cell, CSC; Columella differentiated, Col; Lateral Root Cap, LRC; Epidermis, Epi; Cortex, Cor; Endodermis, En; Vasculature, Vasc. Cortex and Endodermis comprise the ground tissue (green); the columella tissue is represented in orange, and QC cells are yellow. (A) SCR expression domain, in QC, ground tissue stem cells and endodermis (B), and WOX5 expression domain in QC (C). (TIF) [file pbio.1001724.s001.tif]

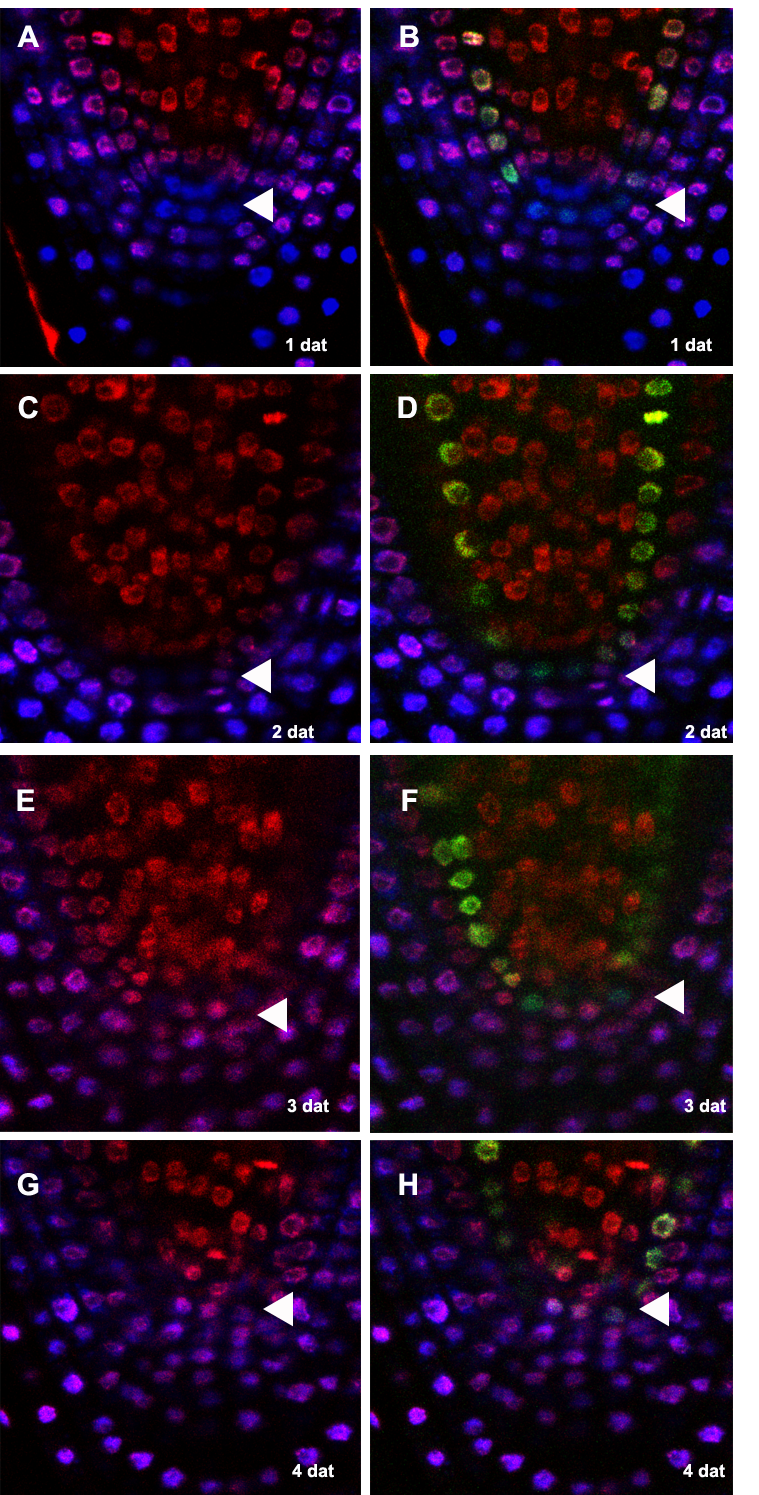

Supplement: Figure S2 — QC incorporates F-ara-EdU at longer times than surrounding stem cells. Left images show red (F-ara-EdU) and blue (DAPI staining) channels; right pictures show overlayed green (pSCR::SCR:GFP) channel. Arrowhead shows QC region that is stained by pSCR::SCR:GFP. Note that all green nuclei have no F-ara-EdU signal at 1–3 dat, but they show signal at 4 dat. Root meristem shown (A–B) 1 dat, (C–D) 2 dat, (E–F) 3 dat, and (G–H) 4 dat. (TIF) [file pbio.1001724.s002.tif]

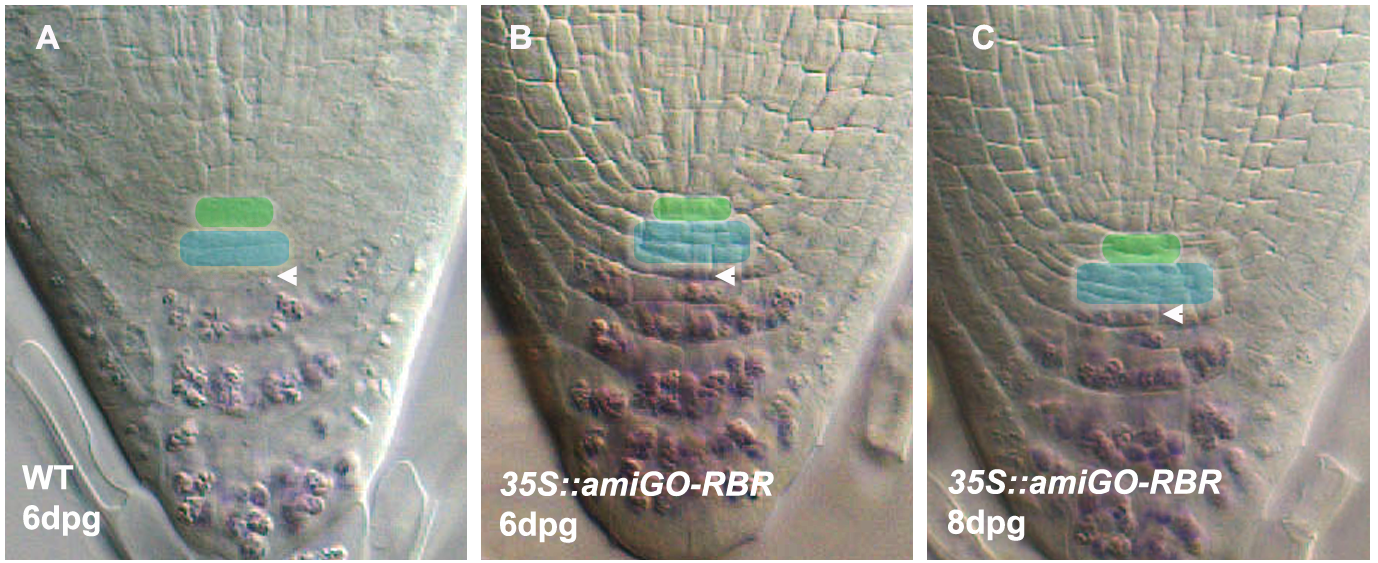

Supplement: Figure S3 — RBR down-regulation induces divisions in the QC and CSCs, leading to extra columella layers. (A) In roots of 6 dpg WT seedlings, a single, or a dividing, CSC (blue colored) is present between the QC (green) and the first columella layer with starch granules (arrowhead). (B) In roots of 6 dpg p35s::amiGO-RBR seedlings, three layers of nondifferentiated columella cells (blue) are present between the QC (green) and the first columella layer with starch granules (arrowhead). (C) In roots of 8 dpg p35s::amiGO-RBR seedlings, divisions of the QC are observed (green) together with extra proliferation of nondiferentiated collumela cells (blue) above first columella layer with starch granules (arrowhead). (TIF) [file pbio.1001724.s003.tif]

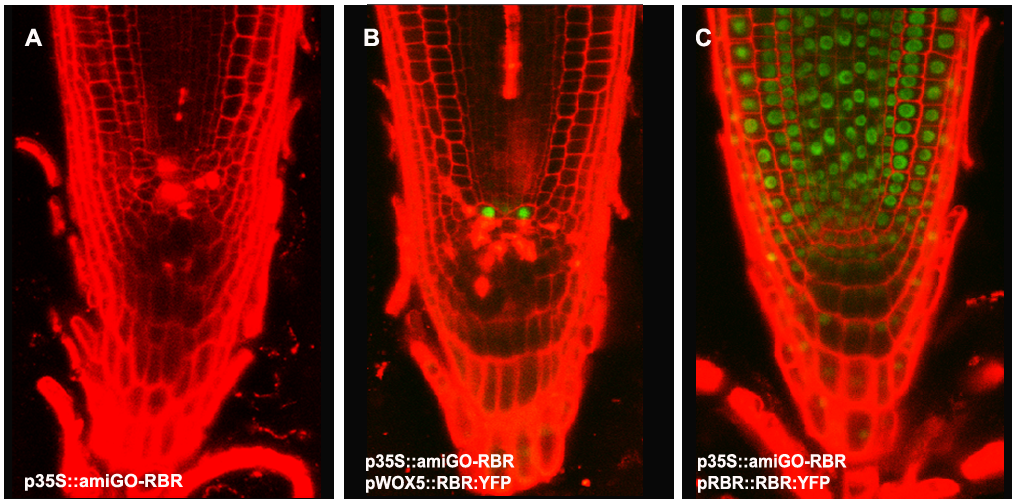

Supplement: Figure S4 — amiGO RBR lines can be complemented by constructs lacking the ami-complementary region. 12 dpg p35S::amiGORBR (A), complemented with pWOX5::RBR:vYFP (B) or pRBR::RBR:vYFP (C), show partial (B) and total (C) complementation of the p35S::amiGORBR phenotype (A). (TIF) [file pbio.1001724.s004.tif]

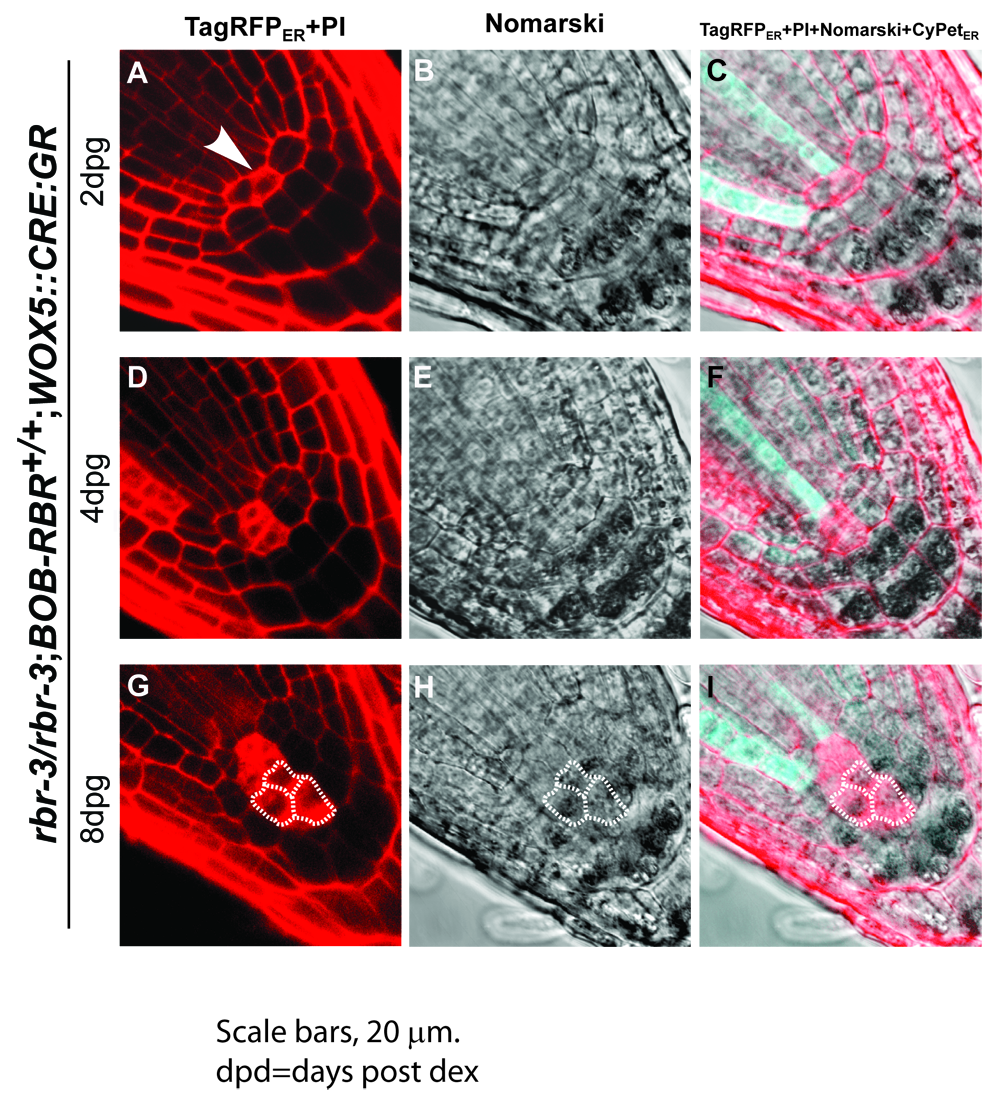

Supplement: Figure S5 — RBR-depleted QC divides and daughters differentiate as mature Columella. CLSM images of a single root tip recorded at 2 (A–C), 4 (D–F), and 8 (G–I) dpg rbr/rbr;BOB-RBR+/+;pWOX5::CRE:GR germinated on Dex-containing medium. A single QC cell (A and C, TagRFPER marked clone) missing one or two RBR copies divides (D and F) and ultimately gives rise to differentiated columella cell marked by starch granules (G to I, three cells enclosed by a dashed line). (TIF) [file pbio.1001724.s005.tif]

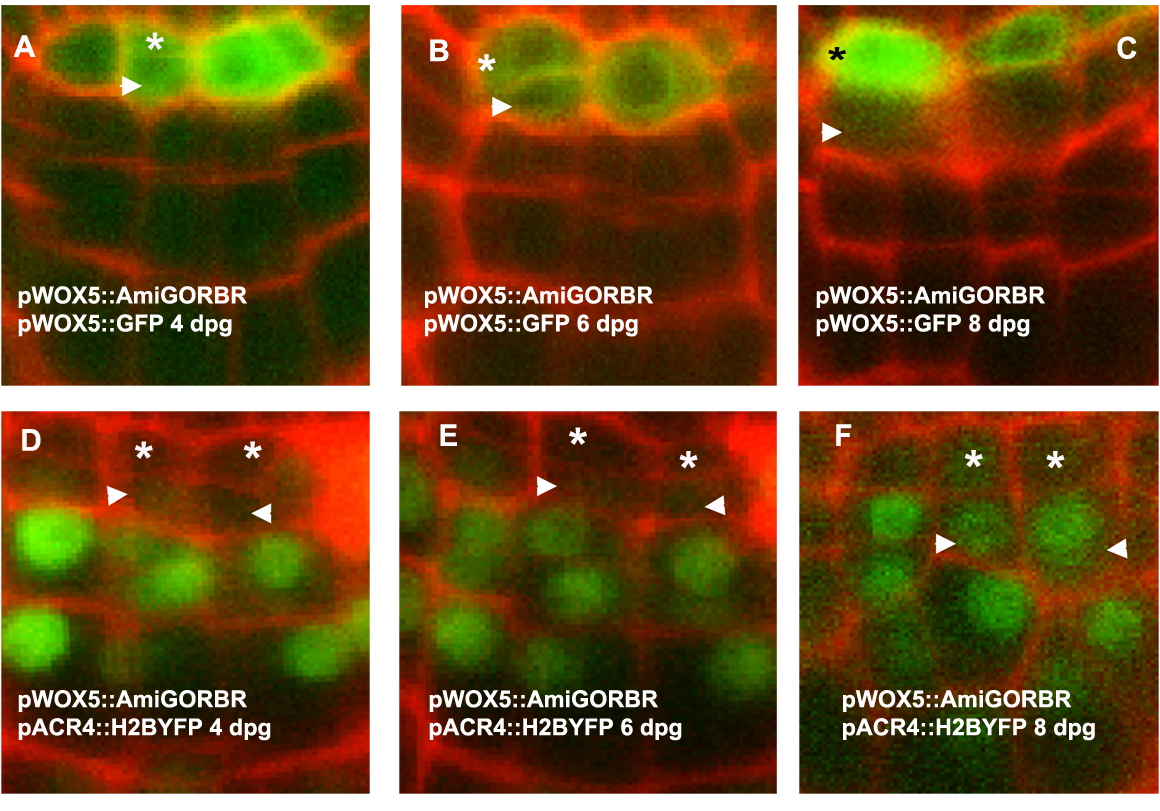

Supplement: Figure S6 — WOX5 and ACR4 marker accumulation before and after QC division. pWOX5::GFP expression (A to C) and pACR4::H2B:YFP (D to F) was monitored and recorded from day 4 until day 8 postgermination in dividing QCs of pWOX5::amiGO-RBR roots. Asterisks indicate shootward daughters and arrowheads point to rootward daughters. (TIF) [file pbio.1001724.s006.tif]

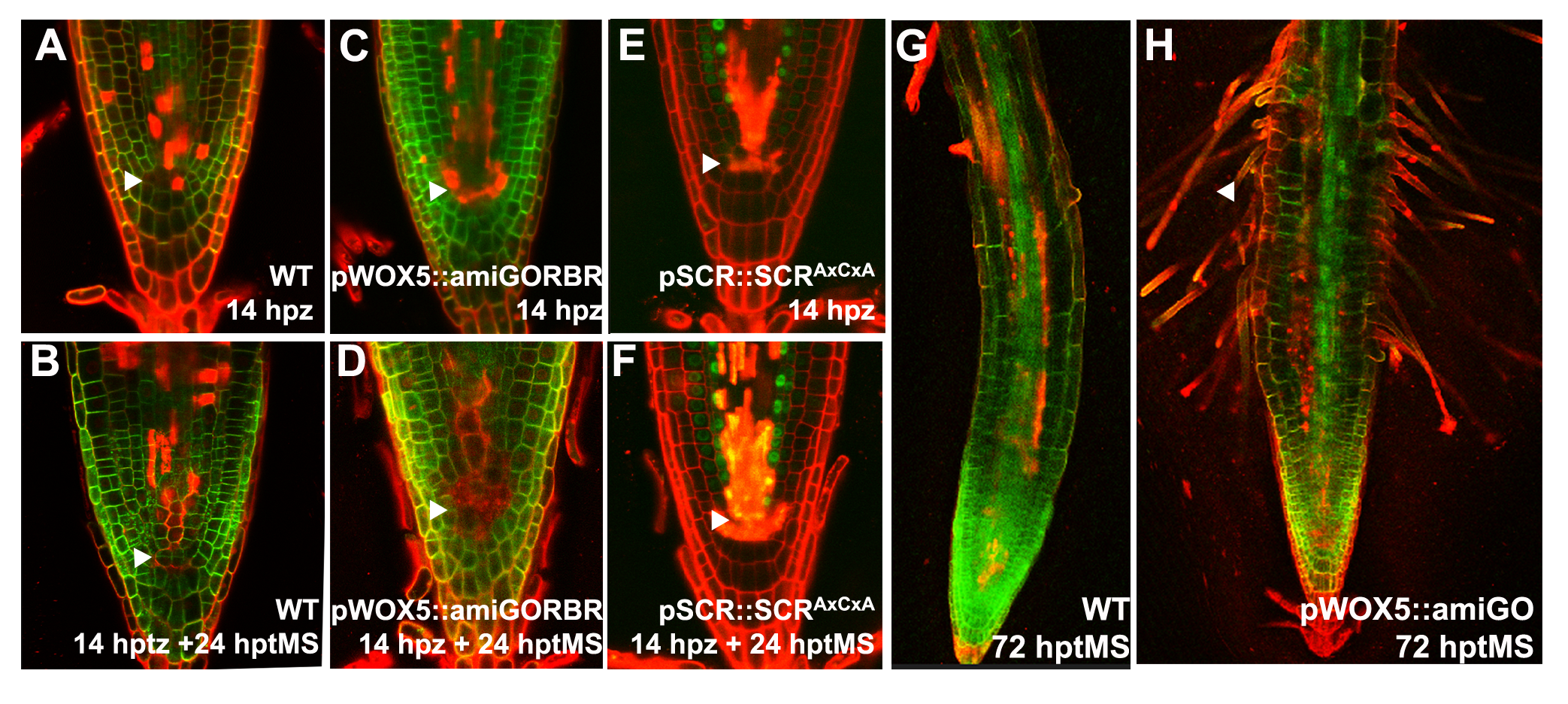

Supplement: Figure S7 — Zeocin effects in the root stem cell niche. 5 dpg seedlings from Col0 and pWOX5::amiGO backgrounds were transferred to medium with or without Zeocin (40 µM) for 14 h (hpz), analyzed, then transferred back to MS medium (hptMS), and monitored at 24 to 72 hptMS CLSM images of root meristems of Col-0 WT (A, B, and G), pWOX5::amiGORBR (C, D, and H), and pSCR::SCRAxCxA:YFP, scr-4 (E and F). (TIF) [file pbio.1001724.s007.tif]

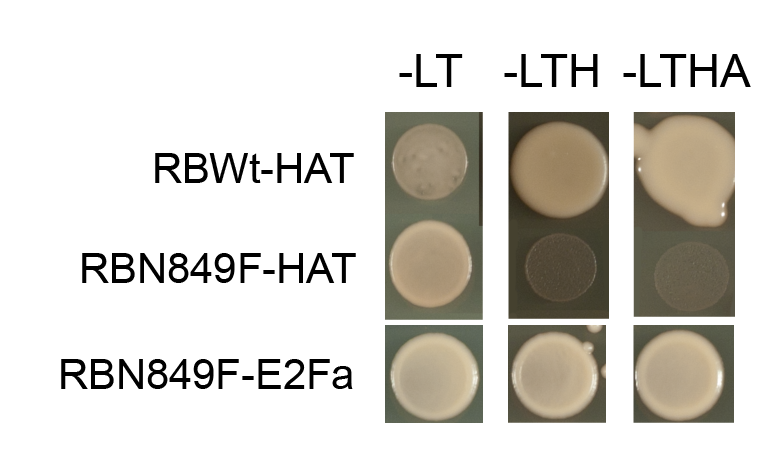

Supplement: Figure S8 — RBRN849F fails to interact with LxCxE-containing proteins. Yeast two-hybrid analysis showing interaction between RBR and HAT2, RBRN849F and E2Fa, and disruption of interaction between RBRN849F and HAT2. (TIF) [file pbio.1001724.s008.tif]
