# Supplementary material for: A SCARECROW-RETINOBLASTOMA Protein Network Controls Protective Quiescence in the Arabidopsis Root Stem Cell Organizer
Source: PLoS Biol. 2013 Nov 26;11(11):e1001724. doi: 10.1371/journal.pbio.1001724 (PMC3841101; doi:10.1371/journal.pbio.1001724)
Supplement: Table S1 — List of plant constructs generated in this study. Plant material used in the study and resistance and reference information. (DOCX) [file pbio.1001724.s009.docx]

| **Constructs** | **Vector (plant resistance)** | **Reference** |
| --- | --- | --- |
| p35S::amiGORBR | pGII229 (pptR) | This paper |
| pRCH1::amiGORBR | pGII229 (pptR) | This paper |
| pSCR::amiGORBR | pGII226 (KanR) | This paper |
| pWOX5::amiGORBR | pGII226 (KanR) | This paper |
| pRCH1::GFP | pGreen (KanR) | (Casamitjana-Martínez et al. 2003) |
| p35S::vYFP:Target | pB7m34GW (pptR) | This paper |
| pWOX5::GFP | pGREENII-0229 (pptR) | (Xu et al., 2007). |
| pSCR::SCR:YFP | pB7m34GW (pptR) | This paper |
| pSMB::SMB:YFP | pGREENII-0229 (pptR) | (Willemsen et al. 2008) |
| pACR4::ACR4:GFP |  | (De Smet et al. 2008) |
| pACR4::H2B:YFP |  | (De Smet et al. 2008) |
| pWOX5::RBD3U:YFP | pGII225 (NorfR) | This paper |
| pWOX5::RBN849FD3U:YFP | pH7m34GW (HygroR) | This paper |
| BOB; HS::CRE | pGII124 (MtxR) | (Wachsman et al. 2011) |
| BOB-RBR, WOX5::CRE:GR | pGII124 (MtxR) | (Wachsman et al. 2011) |

**Table S1. List of plant constructs generated in this study**
