# Supplementary material for: A SCARECROW-RETINOBLASTOMA Protein Network Controls Protective Quiescence in the Arabidopsis Root Stem Cell Organizer
Source: PLoS Biol. 2013 Nov 26;11(11):e1001724. doi: 10.1371/journal.pbio.1001724 (PMC3841101; doi:10.1371/journal.pbio.1001724)
Supplement: Text S1 — Supplementary information. Construction and testing of the AMIGO gene silencing system and supplemental references. (DOC) [file pbio.1001724.s010.doc]

### Supplemental information to:

**Alfredo Cruz-Ramírez^1,2§^, Sara Díaz-Triviño^1,3§^, Guy Wachsman^1‡^, Yujuan Du^1,3‡^, Mario Arteága-Vázquez^4,5^, Hongtao Zhang^1^, Rene Benjamins^1,3^, Ikram Blilou^1,3^,** [**Anne B. Neef**](http://www.pnas.org/search?author1=Anne+B.+Neef&sortspec=date&submit=Submit)**^6^, Vicki Chandler^4^ and Ben Scheres^1,3 *^.**

^1^ Department of Molecular Genetics, Utrecht University, 3584 CH Utrecht, The Netherlands.

^2^ Laboratorio Nacional de Genómica para la Biodiversidad, Cinvestav Sede Irapuato, Km 9.6 Libramiento Norte Carretera Irapuato-León, 36821, Irapuato, Gto., Mexico.

^3^ Current affiliation; Department of Plant Developmental Biology, Wageningen University Research, 6708 PB Wageningen, The Netherlands.

^4^BIO5 Institute and Department of Plant Sciences, University of Arizona, Tucson, Arizona 85721. USA.

^5^Current affiliation: Instituto de Biotecnología y Ecología Aplicada, Universidad Veracruzana, Xalapa; Veracruz, México.

^6^ Institute of Organic Chemistry, University of Zurich, Winterthurerstrasse 190, CH-8057 Zurich, Switzerland

**^§^**These authors contributed equally to this work

**^‡^**These authors contributed equally to this work

* Correspondence:ben.scheres@wur.nl

**Construction and testing of the AMIGO gene silencing system.**

The gametophytic lethality of null alleles is a major constraint on studying loss of RBR function in *Arabidopsis* (Ebel et al. 2004). Previous strategies to overcome this problem have used hairpin RNAi-based technology (hpRNAi) or co-suppresion (Wildwater et al. 2005; Borghi et al. 2010). hpRNAi-mediated gene silencing utilizes small interfering RNAs, which can traffic intracellularly causing non-cell autonomous silencing. . Moreover, these strategies cause degradation of any *RBR* transcript, thus precluding tissue specific complementation of silenced lines. RBR null clones can be produced by a two-color clonal deletion system (Wachsman et al. 2011), but specific complementation is not straightforward in this system. Therefore we developed a cell-type specific silencing system that allows for complementation with point mutant variants of the gene in study, which specifically targets a 21 bp sequence (TS) of the 3’ UTR of the *RBR* mRNA using the miR319 precursor sequence as a template as previously described for other amiRNAs (Schwab, et.al. 2006) (Fig. 2A).

Expression of the amiGO-RBR precursor from the ubiquitous CaMV 35S promoter (*p35S::amiGO-RBR*) leads to aberrant and supernumerary divisions in the stem cell niche, and cell death in the columella root cap and vascular tissue (Fig. 2B,C). Over time, these defects become axacerbated, and extra columella and lateral root cap (LRC) cell layers appear (Fig. 2D). These alterations are similar to those observed in the *pRCH1::RBR* hpRNAi (rRBr) line (Wildwater et al., 2005) and in homozygous *rbr* null clones (Wachsmann et al., 2011), suggesting that amiGO-RBR efficiently silences the *RBR* gene. Accordingly, *RBR* transcript and protein levels are decreased in *p35S::amiGO-RBR* seedlings (Fig. 2E-F), where mature amiGO-RBR is expressed (Fig. 2G). We conclude that mature *amiGO-RBR* in *p35S::amiGO-RBR* seedlings efficiently targets *RBR* transcripts, and produces an increase in stem cell activity as previously described*.*

To detect the *in vivo* activity of the *amiGO-RBR*, we created an *amigo-RBR* sensor comprising a fusion of the target sequence to the vYFP fluorescent reporter gene, driven by the broadly expressed 35S promoter, such that YFP signal will be absent in cells where amiGO-RBR is active (Fig. 2H and methods). *pRCH1::amiGO-RBR* plants express *amiGO-RBR* in transit amplifying cells of the meristem but not in the columella root cap and show strongly reduced sensor signal in the RCH1 domain (Fig. 2J) showing efficient silencing of the target sequence and no distal spreading of the silencing.

*pRCH1::amiGO-RBR* roots display alterations at 8 dpg (data not shown) and 12dpg (Fig. 2L, n=12) that are similar, although less severe, to those observed in *p35S::amiGO-RBR* seedlings (Fig. 2K, n=20). The number of mature root cap cells is however reduced in *pRCH1::amiGO-RBR* in comparison with *p35S::amiGO-RBR* roots, consistent with the absence of RCH1 promoter activity from columella and lateral root cap*.*

To test complementation after silencing by amiGO-RBR, we fused an *RBR* CDS lacking the 3’ UTR (*RBRΔ3U)* to the *vYFP* CDS, under the control of the *RBR* native promoter (*pRBR::RBRD3U:vYFP*). In this line the 3’ UTR, and thus the target sequence (TS) of the *amiGO–RBR* is absent; hence we expect this chimeric *RBR* variant to be resistant to the silencing. Homozygous *p35S::amiGO-RBR;pRBR::RBRD3U:vYFP* (Fig. S4C n=12) and *p35S::amiGO-RBR;pWOX5::RBRD3U:vYFP* roots (Fig.S4B n=7) indeed reveal the promoter-dependent complementation of the phenotypes described above for *p35S:amiGO-RBR* roots (Fig. S4A).

**Supplemental References:**

Borghi L, Gutzat R, Fütterer J, Laizet Y, Hennig L, and Gruissem W. 2010. Arabidopsis RETINOBLASTOMA-RELATED Is Required for Stem Cell Maintenance, Cell Differentiation, and Lateral Organ Production. *The Plant Cell Online* **22**: 1792 –1811.

Casamitjana-Martínez E, Hofhuis HF, Xu J, Liu C-M, Heidstra R, and Scheres B. 2003. Root-specific CLE19 overexpression and the sol1/2 suppressors implicate a CLV-like pathway in the control of Arabidopsis root meristem maintenance. *Curr. Biol.* **13**: 1435–1441.

Ebel C, Mariconti L, and Gruissem W. 2004. Plant retinoblastoma homologues control nuclear proliferation in the female gametophyte. *Nature* **429**: 776–780.

De Smet I, Vassileva V, De Rybel B, Levesque MP, Grunewald W, Van Damme D, Van Noorden G, Naudts M, Van Isterdael G, De Clercq R, et al. 2008. Receptor-Like Kinase ACR4 Restricts Formative Cell Divisions in the Arabidopsis Root. *Science* **322**: 594 –597.

Wachsman G, Heidstra R, and Scheres B. 2011. Distinct Cell-Autonomous Functions of RETINOBLASTOMA-RELATED in Arabidopsis Stem Cells Revealed by the Brother of Brainbow Clonal Analysis System. *The Plant Cell Online* **23**: 2581 –2591.

Wildwater M, Campilho A, Perez-Perez JM, Heidstra R, Blilou I, Korthout H, Chatterjee J, Mariconti L, Gruissem W, and Scheres B. 2005. The RETINOBLASTOMA-RELATED Gene Regulates Stem Cell Maintenance in Arabidopsis Roots. *Cell* **123**: 1337–1349.

Willemsen V, Bauch M, Bennett T, Campilho A, Wolkenfelt H, Xu J, Haseloff J, and Scheres B. 2008. The NAC Domain Transcription Factors FEZ and SOMBRERO Control the Orientation of Cell Division Plane in Arabidopsis Root Stem Cells. *Developmental Cell* **15**: 913–922.

Xu, J., Hofhuis, H., Heidstra, R., Sauer, M., Friml, J., and Scheres, B. (2006). A Molecular Framework for Plant Regeneration. Science *311*, 385–388.
